# The Anti-SARS-CoV-2 Antibody Response in a Centenarian Woman: A Case of Long-Term Memory?

**DOI:** 10.3390/v13091704

**Published:** 2021-08-27

**Authors:** Elisa Toppi, Veronica De Molfetta, Gianpaolo Zarletti, Massimo Tiberi, Paola Bossù, Giuseppe Scapigliati

**Affiliations:** 1 IRCCS Fondazione Santa Lucia, Experimental Neuropsychobiology Lab, via del Fosso di Fiorano 64, 00143 Roma, Italy; p.bossu@hsantalucia.it; 2 Dipartimento Innovazione Biologica, Agroalimentare e Forestale, Università della Tuscia, 01100 Viterbo, Italy; v.demolfetta@gmail.com (V.D.M.); gianpaolo.zarletti@gmail.com (G.Z.); massimo_tib@yahoo.it (M.T.); scapigg@unitus.it (G.S.)

**Keywords:** SARS-CoV-2, COVID-19, in vitro IgG, B cell memory, cell-ELISA

## Abstract

SARS-CoV-2 is the virus responsible for the COVID-19 pandemic, causing respiratory syndrome and other manifestations. The clinical consequences of the SARS-CoV-2 infection are highly heterogeneous, ranging from asymptomatic and mild to severe and fatal conditions, with the highest mortality rate reached among elderly people. Such heterogeneity appears strongly influenced by the host immune response, which in turn is profoundly affected by aging. In fact, the occurrence of a low-grade inflammation and a decline in specific immune defense is generally reported in older people. Although the low ability of B cells to provide primary and secondary specific responses with a consequent increase in susceptibility to and severity of virus infections is generally described in elderly people, we would like to present here the particular case of a 100-year-old woman, who recovered well from COVID-19 and developed a long-term memory against SARS-CoV-2. Following the infection, the patient’s blood was tested with both a classical ELISA and a specific Cell-ELISA addressed to measure the anti-spike S1 specific IgG released in plasma or produced in vitro by memory B cells, respectively. While showing negative on classical serological testing, the patient’s blood was positive in Cell-ELISA up to 1 year after the infection. Our observation highlights a potential mechanism of B cell-dependent, long-term protection in response to SARS-CoV-2 infection, suggesting that in a case of successful aging, the absence of specific antibodies in serum does not necessarily mean the absence of immune memory.

## 1. Introduction

In December 2019, an unknown new Coronavirus causing an infectious respiratory syndrome was observed in Wuhan, China. The new coronavirus was identified as SARS-CoV-2 and, in March 2020, the World Health Organization (WHO) declared a global pandemic caused by this virus. Coronaviruses are a family of enveloped, single-stranded RNA viruses described as early as the 1960s [1]. A key feature of these virulent viruses is their ability to replicate in epithelial cells and pneumocytes of the lower respiratory tract in humans and thus, cause pneumonia and, in severe cases, acute respiratory distress syndrome (ARDS) [2]. In a heterogeneous infectious disease such as COVID-19, host factors are essential in determining the disease severity and progression [3]. Evidence from studies around the world suggests that age is the most significant risk factor for severe COVID-19 disease and its adverse health outcomes [4,5,6,7].

Given the disproportionate burden of severe COVID-19 disease and death in older adults, it is important to understand the mechanisms underlying this age-related vulnerability. The remodeling of the immune system due to aging and immunosenescence are considered to be the main reasons for the increased susceptibility to infections, particularly influenza, and for impaired immune responses to vaccination [8,9]. In particular, the antibody response in the elderly seems to be qualitatively and quantitatively affected and sometimes dysfunctional, leading to the decreased numbers of functional B cells, decreased titers of antigen-specific antibodies, and reduced duration of the humoral response, all together causing an impaired control of viral infections [10].

In contrast to the above-described condition that mainly affects frail subjects and implies COVID-19 high vulnerability, in fewer cases, aging is characterized by high physical, psychological, and social functioning, in the absence of common age-related diseases and disability, known as successful aging. Centenarians are the leading exponent of successful aging, which appears driven by a combination of genetic, immune, and environmental factors [11]. Accordingly, proteome analysis studies have shown that centenarian individuals have a distinct expression of proteins/pathways that reflect healthy immune function, including a preserved humoral immune response and increased B-cell activation [12].

To date, still few studies are available on how aging can modify the host immune response against the novel virus strain SARS-CoV-2. Regardless, there is a growing public health interest in the SARS-CoV-2-infected aged population, and research on successful aging with this virus is highly warranted.

Our paper aims to illustrate a clinical case history of a centenarian woman patient who recovered from COVID-19 pneumonia, developing an immune response characterized by a long-term B cell memory response against SARS-CoV-2.

## 2. Case Description

On the 23rd of February 2020, a 99-year-old female patient experienced major flu symptoms including cold, persistent cough, dyspnea, and a fever at 38 °C. After consultation, the family physician prescribed paracetamol for temperature control. Over the next 2 days, the symptoms worsened and difficulties in respiration and persistent coughing had arisen. The primary care physician performed a home visit and after noting severe dyspnea with a blood oxygen saturation at 90, recommended hospitalization. Thus, on 25 February, the patient went to the ER at Viterbo hospital, in Italy, for dyspnea and cough, where she was admitted at a high level of urgency because of the possible compromise of vital parameters in a short time. She appeared alert, oriented, and with dyspnea. At the objective examination humid bronchial noises were reported. The initial diagnosis was acute asthmatic COPD accompanied by not datable atrial fibrillation. The chest X-ray, however, showed a thickening of the peribronchial and bronchilovascular interstitium, probably due to interstitial disease. However, because of the national indications for SARS-CoV-2 testing strategies that were in force in those days, the SARS-CoV-2 specific PCR swab was not performed. According to the ER medical records, the patient was still administered with antibiotic therapy levoflocxacin, Deltacortene, Lasix, Aerosol with Clenil, Fluibron, Clexane 4000, Pantorc 40 mg and administration of natural water approximately 2 L/day. The arterial blood gas analysis also highlighted a picture of metabolic alkalosis, which, together with the blood tests carried out at the entrance of the ER, showed an increase in the index of inflammation, as evaluated by CRP, D-dimer and LDH measurements. Oxygen therapy with 3 L/min was started. The subsequent blood examinations in the following 2 days showed an initial renal failure, glycemic alteration, and an increase in D-dimer and in inflammatory indexes (ESR, CRP). After 3 days of hospitalization, the patient showed a good response to therapy and her clinical parameters were stabilized with a blood oxygen saturation at 95 and a blood pressure at 148/97. She was discharged to continue her treatment at home with the help of the attending physician. Similar for the admission, and for the same reasons, no SARS-CoV-2 swab was performed at discharge.

Before hospitalization, the centenarian patient was in a good psychophysical status. She did not show cognitive disturbances and had a high level of autonomy with preservation of social and productive activities. After hospital discharge, the patient showed a moderate respiratory fatigue, some dyspnea when walking, and very mild memory problems that did not require a neurologist visit. To date, the centenarian patient enjoys satisfactory clinical conditions, similar to those before the respiratory symptoms and hospital admission.

**Figure 1:**
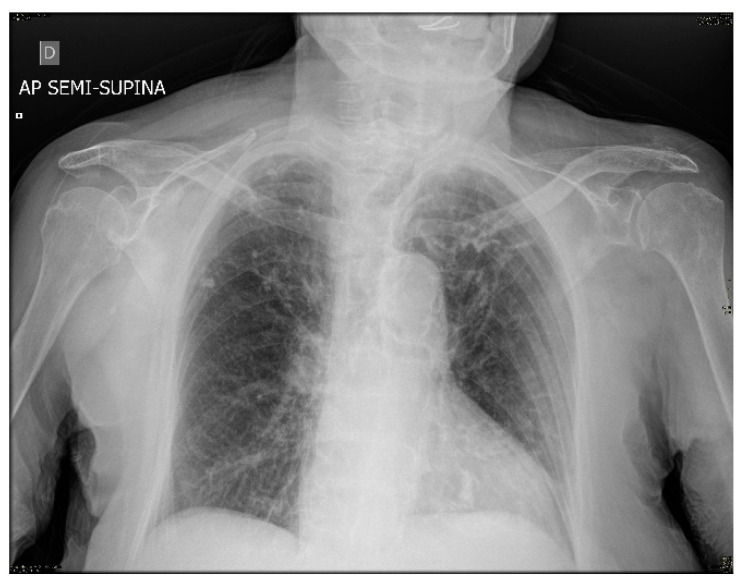
Anterior chest X-ray of the centenarian patient showed a thickening of the peribronchial and bronchilovascular interstitium, probably due to interstitial disease.

Since at the time of the hospital admission, in Italy, the COVID-19 pandemic was still in its infancy and the molecular swabs for SARS-CoV-2 were not available in Viterbo. The hospital health care providers did not deem it appropriate to send the patient to a hospital of a different city for the molecular swab. However, later on, a lung specialist released a medical report based on the hospital medical records, showing an image compatible with SARS-CoV-2 infection, as suggested by chest X-ray results (Figure 1).

Afterwards, the patient underwent blood sampling twice, exactly 137 (T1) and 332 (T2) days after the symptoms’ onset, to measure the anti-SARS-CoV-2 spike S1 specific IgG antibodies in both plasma and PBMC culture. Briefly, the levels of plasma IgG were measured by a commercial ELISA kit using an adsorbed recombinant S1 peptide (Dia.Pro), while the method of the Cell-ELISA assay addressed to evaluate the IgG produced in vitro against spike-S1 viral protein using 10^6^ PBMC/well was previously described in detail [13] and here applied without modifications. Briefly, the Cell-ELISA assay is based on the measurement of specific IgG secreted in vitro by PBMC co-cultured with the immunization antigen. Overall, by combining the ELISA and Cell-ELISA it is possible to evaluate at the same time and in the same donor the presence of plasma antibodies and the presence of antibodies secreted by memory B cells, providing the possibility to detect a longlasting humoral response after immunization/vaccination, even when circulating IgG declined and became undetectable in the blood. The difference between the ELISA and Cell-ELISA methods, their sensitivity/threshold, and limitations, including possible false positive results, have been previously reported [13].

In parallel, anti-SARS-CoV-2 spike S1 specific IgG antibodies both in the plasma and in the PBMC culture were measured in an additional group of six, aged female subjects that received naso-pharyngeal swab screening less than 4 months before the blood withdrawal. Among those, three subjects resulted negative (mean age 75 ± 4.35 years) and three subjects resulted positive (mean age 86 ± 2.88 years) in SARS-CoV-2 RT-PCR and, accordingly, in ELISA and Cell-ELISA, acting as negative and positive controls for the assays, respectively. More specifically, OD_450_ = 0.20 ± 0.005 were the values obtained for plasma IgG levels in the three patients negative for SARS-CoV-2 (used as negative controls in ELISA), and OD_450_ = 1.9 ± 0.78 in the three positive patients (used as positive controls in ELISA). Likewise, regarding Cell-Elisa, the mean of values obtained in the SARS-CoV-2 negative patients were OD_450_ = 0.02 ± 0.012 (used as negative controls in Cell-ELISA), and OD_450_ = 0.27 ± 0.014 for the positive subjects (used as positive controls in Cell-ELISA).

## 3. Results

**Figure 2:**
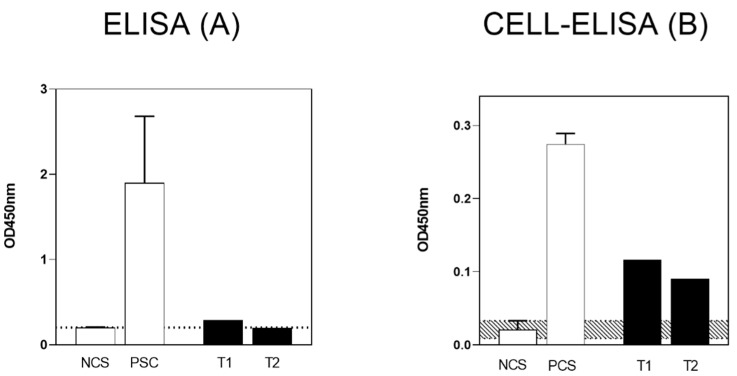
Measurement of anti-SARS-CoV-2 spike S1 specific IgG antibodies both in the plasma by ELISA (**A**) and in PBMC culture, by Cell-ELISA (**B**) of Negative Control Subjects (NCS) and Positive Control Subjects (PCS) and of the centenarian patient at the two time points (T1 and T2). The dotted area in A and the shaded area in B panel indicate the cut-off OD_450_ value discriminating between negative and positive control values, calculated as the mean OD_450_ value ± 2 SD of negative control subjects, in each condition.

When the ELISA serological examination was performed in the centenarian woman’s plasma, no measurable circulating IgG were observed at both time points (day137: OD_450_ = 0.23 and day332: OD_450_ = 0.20). The serological negative results were evident by comparison with negative (OD_450_ = 0.20 ± 0.005) and positive (OD_450_ = 1.9 ± 0.78) control patients (Figure 2, left panel), and confirmed by the cut-off value of the commercial ELISA kit considering positive the samples with OD_450_ > 1.1, as reported in the previous publication [13].

At the same time points of ELISA, the Cell-ELISA test was performed on isolated PBMC as described [13], and the absorbance net values after background subtraction were OD_450_ = 0.12 on day 137 and OD_450_ = 0.09 on day 332 (Figure 2, right panel). Such values can be considered both positive, since laying far above negative control values (OD_450_ = 0.02 ± 0.012), though below positive control values (OD_450_ = 0.27 ± 0.014, respectively) and as referred to the previous publication, where the threshold value discriminating positive vs. negatives was OD_450_ > 0.06 [13].

## 4. Discussion

The clinical case here described regards a centenarian woman without previous medical illness and disability, who was very likely infected with SARS-CoV-2 in late February 2020, as strongly suggested by the symptoms presented at her hospital admittance and by the later report released by a respiratory specialist confirming a condition compatible with COVID-19 pneumonia (Figure 1). Regardless of her very advanced age, the patient recovered well from COVID-19 and her anti-virus antibody response was taken into account. Specifically, IgG antibodies directed against SARS-CoV-2 spike S1 were evaluated twice and up to 1 year after the symptoms’ onset. The measurement of circulating specific antibodies was paralleled by the evaluation of specific antibodies released in vitro by memory B cells (Figure 2). The main finding of this report is that while the serological analyses performed more than 4 months up to 1 year after infection were negative, the Cell-ELISA data were positive and fairly constant over time, suggesting a long lasting immune memory linked to B lymphocytes. This finding was confirmed when the values obtained from the centenarian patient’s samples were compared with samples from control aged subjects, either negative or positive for virus swab and for both serology and cell-ELISA (Figure 2), highlighting that the observed memory response in the patient is in line with a previous SARS-CoV-2 infection.

These data suggest that, after infection or following vaccination, even when circulating IgG are declined, the immunological memory may offer good protection against re-infections, protecting at least against severe symptoms, since IgG against SARS-CoV-2 are already detectable at 2–4 days [14].

Older adults are highly vulnerable to COVID-19 and the age-related impairment of the immune defense against virus infection and immunosenescence have been proposed among the causes of the increased risk of severe disease and mortality. Accordingly, aged people, as compared to younger subjects, have an antibody response different in quality and quantity and sometimes dysfunctional with decreased numbers of functional B-cells secreting immunoglobulins, in correlation with decreased titers of antigen-specific antibodies. Although circulating memory B cell numbers and antibodies are expected to increase proportionately with aging, they may contribute to dysfunctional responses to new infections. Specifically, the aging aberrant antibody responses to new viruses may regard decreased affinity maturation, poor neutralization of antigens, as well as heightened production of antibodies prone to autoreactivity [15]. In addition, these elderly B cells escape apoptosis, leading to the accumulation of abnormal antigen- and cytokine-producing cells [16]. The general consequence of this condition may be a reduced duration of the humoral response with a low ability of B cells to provide primary and secondary specific responses and a consequent increase in susceptibility to and severity of virus infections. Within such a scenario, considering the immune response of centenarians to SARS-CoV-2 might be a valuable approach to investigating the COVID-19 vulnerability in aging. In fact, centenarians are likely to achieve successful aging attributable to mechanisms linked to unique genetics, probably resulting from the interaction between mtDNA, microbiome, and nuclear DNA, environment, and any somatic mutations that occur during aging [11]. Indeed, studies across age regarding SARS-CoV-2-specific persistence, tissue specificity and inflammatory response triggering, as well as the role of immune memory cells, are mainly unknown to date, presenting a still unexplored area for studying COVID-19 pathogenesis [10].

Of course, some limitations occur in this study. As described, the lack of the SARS-CoV-2 molecular test in the centenarian patient was a weakness, though strong evidence suggests that the patient was indeed affected by COVID-19, as indicated by post facto diagnosis. In addition, the data obtained with the Cell-ELISA method will certainly need to be expanded in the future by increasing the number of samples analyzed from SARS-CoV-2 infected and control subjects of different age, as well as in the frail versus the healthy elderly. Similarly, the length of time running between immunization or infection and Cell-ELISA analysis should be considered more carefully to better define the antibody memory response duration. A final and more intriguing issue that certainly needs to be investigated in specific follow-up studies is the relationship existing between the PBMC ability to release in vitro anti-Spike S1 IgG and specific anti-viral immune protection. However, the proposed approach to use Cell-ELISA in the context of the present case description can improve the conventional plasma or serum-based ELISA, especially when IgG decline over time, becoming undetectable in serological analysis. In particular, we propose that the presence of the IgG response we have observed in the centenarian patient’s PBMCs may be linked to the successful pathology outcome, in spite of the advanced age of the patient. However, whether the memory antibody response we observed is due to a previous encounter with the virus, enabling a long lasting protection that attenuated the COVID-19 outcome in the centenarian patient, or is rather the consequence of a first infection (the more probable option based on the pandemic development), is impossible to discriminate.

Overall, the present case report regarding a centenarian woman who recovered from COVID-19 suggests that understanding the anti-SARS-CoV-2 memory B cell response in the context of successful aging may help in the assessment of immunization status through the detection of an S1 protein, concurring in identifying individual factors related to facing the COVID-19 pandemic.

Eventually, the Cell-ELISA test we described can become a useful diagnostic tool to detect people who have had infections, but which were negative for serology, to identify false negative patients, especially among older people, in determining which individuals will benefit more than others from vaccination, and to improve the evaluation of the effectiveness of the vaccination campaign currently in progress.

## Data Availability

Data supporting reported results can be found in author’s archives and cand be freely obtained upon request.
